# Case report: Teat stenosis in a suckler cow

**DOI:** 10.3389/fvets.2023.1199021

**Published:** 2023-12-05

**Authors:** Melanie Schären-Bannert, Alexander Starke, Teja Snedec, Laura Vogel, Romy Wagner, Tilman Kühn, Lilli Bittner-Schwerda

**Affiliations:** ^1^Clinic for Ruminants and Swine, Faculty of Veterinary Medicine, Leipzig University, Leipzig, Germany; ^2^Tierarztpraxis Hildebrandt, Mülsen, Germany

**Keywords:** theloscopy, teat surgery, beef cattle, mastitis, milking

## Abstract

Bovine veterinarians are regularly confronted with teat lesions in cows. The number of studies on the diagnosis and treatment of teat lesions as well as the exchange of practical experience among clinicians are extensive in dairy cows compared with suckler cows. The aim of this case report was to describe the successful treatment of teat stenosis in a suckler cow and discuss possible challenges. A four-year-old Simmental cow, in her third lactation and 4 days in milk, was referred to our clinic along with her calf because of teat stenosis in the front left quarter. The owner had repeatedly used a rigid teat cannula in an attempt to relieve the stenosis during the previous lactation. However, the cow had refused to allow the current calf to suckle the affected teat and resisted attempts by the owner to cannulate the teat. The results of clinical examination, ultrasonography, and milk sampling showed stenosis of the proximal, middle, and distal parts of the front left teat cistern, accompanied by thelitis and cisternitis and mild chronic clinical mastitis. Based on published recommendations, treatment of the thelitis, cisternitis, and mastitis was initiated before resolution of the stenosis surgically. The first week of treatment included the administration of an intramammary product containing cefapirin and prednisolone, a systemic non-steroidal anti-inflammatory drug, a wax teat-boogie, and bandaging of the teat. Thereafter, the treatment was reduced to insertion of a wax-teat boogie and bandaging. Conservative treatment resulted in resolution of the mastitis, cisternitis, and stenosis in the proximal and middle parts of the teat, which had most likely been caused by repeated cannulation of the teat by the owner. Lateral theloscopy was then used to remove the distal stenosis, which was the primary lesion. Healing of the surgical wound and resolution of the swelling occurred several days postoperatively, and the calf’s first attempt to suckle the teat was successful. The cow and calf were discharged from the clinic 2 weeks after surgery. A follow-up visit 4.5 months after surgery revealed that the calf was still nursing the teat and the operated quarter was producing a normal amount of milk.

## 1 Introduction

Teat lesions are commonly encountered in bovine veterinary practice and remain a challenge to treat in the field (1). The success rate is substantially affected by the method of treatment, delays between occurrence and treatment, as well as postoperative management (2, 3). Classification of teat lesions (4, 5), diagnostic tools including ultrasound, and sophisticated treatment methods such as theloscopic surgery (3, 6, 7) have been developed for dairy cows. In addition, practical reports concerning teat injuries and their treatment are available to practitioners in the field (1, 8). In contrast, similar information concerning suckler cows is scant (google scholar search, 30.09.2022, keywords: teat, stenosis, suckler cow, beef cattle, cow, cattle, no corresponding publications found). Furthermore, unlike dairy cows, a number of challenges may be encountered when treating teat lesions in suckler cows. They include the calf interfering with wound healing by removing the bandage and sucking the teat and the infeasibility of monitoring and controlling the onset of milk withdrawal postoperatively using a milking machine. Reasons for the latter include neophobia, resistance on the part of the cow, and/or logistical problems. The calf must instead be used to stimulate the gland to allow milk let-down.

The present case report describes the successful treatment of teat stenosis in a suckler cow. Our aim was to lead the discussion on this topic and provide bovine practitioners with our initial experience.

## 2 Case description

### 2.1 Patient information

A Simmental cow (age: 4 years, 3rd lactation) with a suckler calf (age: 4 days) was referred to the Clinic for Ruminants and Swine, Faculty of Veterinary Medicine, Leipzig University, in December 2021 because of teat stenosis. The cow originated from a herd with four other cows and one heifer, which were housed on pasture from spring to autumn and kept indoors on hay and grass silage throughout the winter. The owner reported that stenosis of the front left teat had been present for the last lactation. The problem had been managed by cannulation of the teat with a permanent rigid teat cannula which allowed milk removal once daily and by allowing the calf to suckle the damaged teat (timeline in Table 1). The cow had calved 4 days before admission and had kicked the newborn calf whenever it tried to suck the affected teat. This behavior progressively worsened, and the cow did not allow hand milking or the calf to suckle any of the teats. Based on the history of chronic teat stenosis, the primary care veterinarian decided to refer the cow to our clinic without treatment.

**Table 1:** Timeline of the case.

Timepoint	Event
First lactation	Stenosis in front left quarter, managed by cannulation by owner
Day −4	Calving of cow into second lactation
Day −4–0	Cow kicking newborn, not allowing to suckle or hand milking any of the teats
Day 0	Admission to clinic with calf, diagnosis of stenosis of the proximal, middle, and distal parts of the front left teat, accompanied by thelitis, cisternitis, and mild chronic clinical mastitis
Day 0–7	Initial treatment of the thelitis, cisternitis, and mastitis: administration of an intramammary product containing cefapirin and prednisolone, a wax teat-boogie, and bandaging of the teat (twice daily), systemic non-steroidal anti-inflammatory drug (every other day)
Day 7–36	Reduction of treatment to insertion of a wax-teat boogie and bandaging (twice daily)
Day 36	Lateral theloscopy to remove the distal stenosis (primary lesion)
Day 36–41	Treatment continued with insertion of a wax-teat boogie and bandaging (twice daily)
Day 41–46	Clinical mastitis of affected quarter: administration of an intramammary product containing cefalexin and kanamycin (daily), and a systemic non-steroidal anti-inflammatory drug (single dose)
Day 47	Calf’s first attempt to suckle the teat successful
Day 52	Discharge from the clinic of mother and calf
4.5 months after surgery	Follow-up visit: calf was still nursing the teat and the operated quarter producing a normal amount of milk

### 2.2 Clinical findings

The cow was in good physical condition, and the clinical examination (9) showed no abnormalities for that stage of lactation (4 days in milk). Adspection of the front left quarter showed signs of atrophy of the parenchyma and hypertrophy of the teat. Palpation revealed that the parenchyma had a coarse-grained structure and the front left teat had hardening of the teat wall (doughy texture) with two palpable tissue proliferations involving the mucosa. The first proliferation was an approximately 2.0 cm × 0.5 cm × 0.5 cm elongated structure, which ran from the teat cistern, passed the annular folds, and ended at the gland cistern. The second proliferation was an approximately 0.5 cm × 0.5 cm pea-shaped structure located in the distal part of the teat at the rosette of Fürstenberg. The ostium between the teat and gland cistern, surrounded by the annular folds, was narrower than those of the other quarters. Hand milking of the front left teat was difficult, yielded only a spray of milk compared with the other teats, and elicited severe pain. The milk contained small flakes, and the California mastitis test (CMT) was positive in all four quarters (colostral milk). A total of 20 IU of oxytocin (Oxytocin 10 IU/mL, Serumwerk Bernburg AG) was administered subcutaneously and the cow was milked by hand. The milk in the affected quarter was drained using a cannula (Milking tube, Bovivet, Kruuse, Langeskov, Denmark). Only about 300 mL of milk was produced from the affected quarter compared with 1–2 L in the other quarters, which confirmed parenchymal atrophy.

### 2.3 Diagnostic assessment

Blood samples were collected from the external jugular vein for hematological and clinical chemistry analyses. The latter included determination of the concentrations of magnesium, calcium, phosphorus, sodium, potassium, chloride, total protein, albumin, bilirubin, urea, and creatinine, and the activities of aspartate-aminotransferase, gamma-glutamyltransferase, glutamate dehydrogenase, and creatine kinase (Table 2). The values were considered unremarkable for the respective stage of lactation.

**Table 2:** Haemogram and clinical chemistry traits.

Trait^1^	Unit	Reference^2^	
*Hemogram*			
Leucocytes	G/L	5–10	8.0
Erythrocytes	T/L	5–10	5.98
Hemoglobin	mmol/L	5.5–8.1	7.2
Hematocrit	L/L	0.24–0.46	0.31
MCV	fl	45–65	52.5
MCH	fmol	0.9–1.5	1.20
MCHC	mmol/L	16–21	22.78
Thrombocytes	G/L	100–600	482
*Minerals/Electrolytes*			
Mg	mmol/L	0.90–1.32	0.69
Ca	mmol/L	2.00–2.54	2.41
P	mmol/L	1.55–2.29	1.51
Na	mmol/L	135–157	144
K	mmol/L	3.9–5.2	4.24
Cl	mmol/L	95–110	98.8
*Proteins/Metabolism*			
TP	g/L	68–82	72.1
Alb	g/L	30–39	35.8
Bili	μmol/L	(3.3)–5.3	3.6
Urea	mmol/L	2.0–6.8	2.95
Crea	μmol/L	55–150	145
GT^3^	min	>15 min	> 15 min
*Enzymes*			
AST	U/L	<80	96.9
GGT	U/L	<50	28.2
GLDH	U/L	5–30	9.9
CK	U/L	<200	179

1 Alb, albumin; AST, aspartat-aminotransferase; Bili, bilirubin (total); Ca, calcium; CK, creatinkinase; Cl, chloride; Crea, creatinine; GGT, gamma-glutamyltransferase; GLDH, glutamyldehydrogenase; K, potassium; MCH, mean corpuscular hemoglobin; MCHC, mean corpuscular hemoglobin concentration; MCV, mean corpuscular volume; Mg, magnesium; Na, sodium; P, phosphorus; TP, total protein.

2 Reference values of the Laboratory of Large Animal Clinics, Faculty of Veterinary Medicine, University of Leipzig, chosen according to Kraft and Dürr (10).

3 GT, Glutaraldehyde-Test, reference according to Doll et al. (11).

Ultrasonographic examination (MyLab™One Vet, Esaote Europe BV, Maastricht, The Netherlands) of the udder parenchyma was carried out using a 3.5 Mhz convex transducer. The teat was assessed using a 10.0 Mhz linear transducer with and without the teat immersed in water in a plastic cup (3, 6). The tissue proliferations in the teat were visible as hyperechogenic structures; the size and location are described above. The teat wall was enlarged and swollen as evidenced by increased echogenicity of the teat wall. It was assumed that the distal stenosis had been present in the previous lactation and cisternitis and tissue proliferation in the middle part of the teat were the result of frequent cannulation using a rigid cannula.

Bacteriological culture of the milk samples yielded no growth in all quarters. Therefore, the mastitis could not be further classified and chronic non-specific mastitis was diagnosed according to the guidelines of the German Veterinary Medical Association (12). The final diagnosis was stenosis of the proximal, middle, and distal parts of the front left teat, accompanied by thelitis, cisternitis, and mild chronic clinical mastitis.

### 2.4 Therapeutic intervention

The therapeutic plan was to treat the thelitis, cisternitis, and mastitis first before resolving the stenosis surgically, as recommended by Starke et al. (8) and Geishauser et al. (13). An intramammary product containing cefapirin (300 mg) and prednisolone (20 mg; Mastiplan® LC, MSD, Intervet Deutschland GmbH, Unterschleißheim, Germany) was administered. This was followed by insertion of a wax-teat bougie (Thelasel, selectavet, Dr. Otto Fischer GmbH, Weyarn, Germany) and application of a bandage with an iodine-containing ointment (100 mg/kg povidone iodine, Vet-Sept Salbe, Livisto, aniMedica GmbH, Senden, Germany). This conservative treatment was repeated twice daily with the goal of decreasing the inflammation and swelling and to determine whether milk production in the quarter would return to normal. Meloxicam (0.5 mg/kg meloxicam, Meloxidyl 20 mg/mL, Ceva Tiergesundheit GmbH, Düsseldorf, Germany) was administered every other day to control pain and inflammation. The calf suckled the three remaining teats and did not disturb the bandage.

After 2 days of treatment, the milk was macroscopically normal. The swelling and sensitivity to palpation of the teat had decreased significantly after 7 days. It was therefore decided to discontinue the intramammary and systemic treatments but continue with daily draining of the quarter with a teat cannula followed by placement of a wax-teat bougie and bandage. The demeanor and appetite of the cow remained normal throughout the treatment period.

Monitoring of treatment by daily palpation of the teat showed that the inflammation and stenosis in the proximal and middle parts of the teat slowly decreased after 3 weeks and only chronic indurative cisternitis remained. The milk production of the quarter increased to 1.5–2 L per day (measured by collection of milk at cannulation), which was comparable to the other quarters. However, the distal stenosis, which was approximately 2–4 mm in diameter, could still be palpated. A decision to surgically correct the stenosis via endoscopic surgery was made on day 36 after admission.

High epidural anesthesia using 0.1 mg/kg 2% xylazine (Xylazin 20 mg/mL as xylazine hydrochloride, Serumwerk Bernburg AG, Bernburg, Germany) followed by 25 mL 0.9% sodium chloride (Serumwerk Bernburg AG) was carried out. The cow received 0.5 mg/kg meloxicam and 10 million IU of penethamate hydroiodide (Ingel-Mamyzin®, Boeringer Ingelheim Vetmedica GmbH) at the same time as the epidural anaesthesia. The penethamate hydroiodide was continued for 2 more days at 5 million IU per day. The cow was placed in lateral recumbency on a tilt table approximately 10 min after the epidural injection, when loss of tail tone and the onset of sedation were observed, as described by Kaiser and Starke (2).

The surgical field was aseptically prepared (isopropanol 70%, Dr. Schumacher GmbH, Malsfeld, Germany; Vet-Sept® solution 10%, aniMedica GmbH, Senden-Bösensell, Germany), and lateral theloscopy was carried out to facilitate removal of the granulation and scar tissue at the rosette of Fürstenberg. This was done using a stenosis cutter, as described by Eisenhut. In addition, a Danish model teat knife was used to cross-slit any remaining scar tissue at the rosette to avoid postoperative stricture [H. Haupter and Richard Herberholz GmbH & Co. KG, Solingen, Germany; (2)]. Oxytocin (20 IU) was then administered intravenously, and the teat was observed for passive milk flow. Placement of a wax-teat bougie, bandaging, and daily canulation to drain the milk were continued (2).

Clinical mastitis of the affected quarter characterized by large flakes in the milk and a strong positive CMT score was diagnosed 5 days postoperatively. The quarter was not swollen, and the demeanour of the cow was normal. Treatment consisted of one dose of meloxicam and intramammary treatment with cefalexin and kanamycin (200 mg cefalexin, 100,000 IE Kanamycin; Ubrolexin®, Boehringer Ingelheim Vetmedica GmbH, Rohrdorf, Germany) once daily for 5 days.

The surgical wounds created by the lateral endoscopic approach healed without complications. The mastitis had resolved 11 days postoperatively, and the calf was then allowed to nurse the teat with the goal of stimulating milk production in the quarter. The calf suckled successfully on the first attempt, and nursing was monitored for another 5 days before the cow and calf were discharged from the clinic.

### 2.5 Follow-up and outcomes

The owner was advised to involve the primary care veterinarian immediately for similar cases because repeated cannulation of the teat was the most likely cause of the cisternitis and stenosis in the proximal and middle parts of the teat (14). Had the lesion been restricted to the distal stenosis, hospitalization would have been substantially shorter and the only requirement would have been the endoscopic surgery.

**Figure 1:**
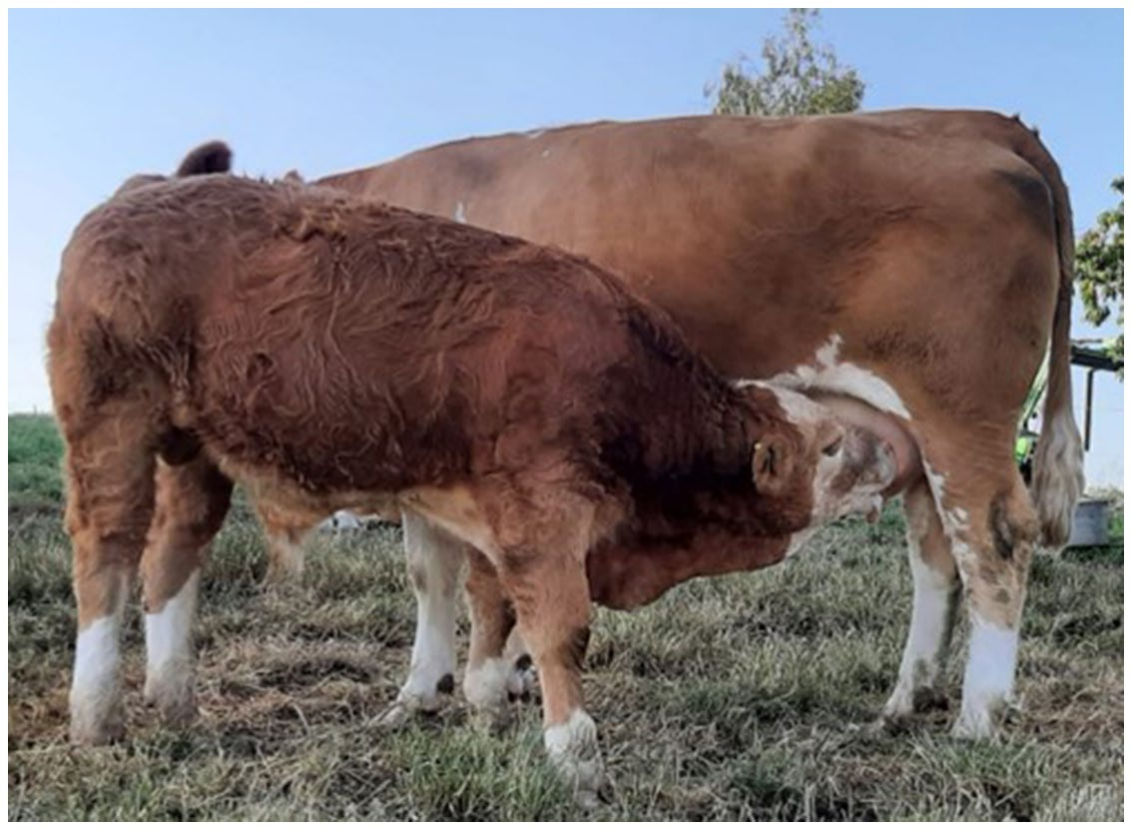
A four-year-old Simmental cow, in her second lactation, with a six-month-old suckler calf. The image shows the pair 4.5 months after successful treatment of teat stenosis.

The primary care veterinarian and the owner remained in close contact with the clinic and reported no changes. The calf had not been weaned 4.5 months after discharge from the clinic (Figure 1), and the quarter was producing a normal amount of milk.

## 3 Discussion

Most teat stenoses appear to be caused by trauma that results in mucous membrane swelling and formation of granulation tissue with subsequent obstruction of the teat canal at the rosette of Furstenberg (7, 15, 16). In dairy cows, risk factors include high milk production associated with a large and sometimes pendulous udder, poor hoof care, and stall-designs that favor teat trauma, which may be self-inflicted or caused by another cow (8, 17). It is believed that blunt trauma to the teat end, which may occur when the teat is stepped on, results in disruption of the integrity of the teat canal causing obstruction (8, 16). In beef cows, reasons for teat injuries are less well documented but traumatic causes are also likely. An observational study by Cooper et al. (18) described risk factors for traumatic and non-traumatic lesions in a flock of suckler ewes. Huntley et al. (19) demonstrated that teat injuries have a significant negative impact on the daily weight gain of suckler lambs, but this cannot be extrapolated to include suckler cows.

The therapeutic approach used in this case was successful and hinged on resolving the thelitis, cisternitis, and mastitis before surgical treatment of the stenosis. Equally important were the use of a lateral theloscopic approach in combination with slitting of the teat canal and allowing the calf to suckle the teat once healing was complete. This is in agreement with a study by Kiossis et al. (20), which showed that the long-term udder health and milkability after teat stenosis were influenced by many factors, including location of the lesion, mastitis, and previous and postoperative treatments.

This case also showed that placement of a rigid teat cannula carries a high risk of complications, such as chronic irritation of the cisternal mucosa, leading to thelitis and cisternitis. A flexible rather than a rigid cannula should be used when repeated drainage of milk is required, although ascending infection leading to mastitis is a risk (14).

To the authors’ knowledge, this is the first report describing the treatment of teat stenosis in a suckler cow. This case was challenging because postoperative treatment excluded the use of a milking machine and thus stimulation of the gland with subsequent milk let-down relied on the calf nursing. This strategy showed that sucking by the calf was gentle enough to allow for simultaneous healing of the teat.

Sharing similar experiences on the treatment of teat lesions in suckler cows is encouraged. The severity and location of lesions will vary and play a role in the success rate of treatment. Additional studies are needed to further knowledge in this field.

## Data availability statement

The original contributions presented in the study are included in the article/supplementary material, further inquiries can be directed to the corresponding author.

## Ethics statement

Ethical approval was not required for the study involving animals in accordance with the local legislation and institutional requirements because this is a clinical case report where procedures were performed with clinical indication and no experimental procedures were done. Written informed consent was obtained from the participants for the publication of this case report.

## Author contributions

MS-B: case work-up, writing of manuscript. AS: patient examination and treatment (including operation), assistance in writing manuscript. TS: patient examination and treatment. LV: patient examination and treatment. RW: referring veterinarian, communication with owner and clinic, patient follow-up. TK: bacteriological examination of milk samples. LB-S: monitoring of patient examination and treatment, assistance in writing manuscript. All authors have read and approved the final version of the manuscript.
